# Comparison of the survival outcomes of laparoscopy versus laparotomy in treatment of early-stage ovarian cancer: a systematic review and meta-analysis

**DOI:** 10.1186/s13048-021-00793-1

**Published:** 2021-03-16

**Authors:** Qingduo Kong, Hongyi Wei, Jing Zhang, Yilin Li, Yongjun Wang

**Affiliations:** 1 Department of Obstetrics and Gynecology, Peking University International Hospital, Life Park of Zhongguancun, Changping District, Beijing, 102206 China; 2 Clinical Medical College, Weifang Medical University, Weicheng District, Weifang, 261000 Shandong China

**Keywords:** Laparoscopy, Laparotomy, Ovarian cancer, Prognosis, Overall survival

## Abstract

**Background:**

Laparoscopy has been widely used for patients with early-stage epithelial ovarian cancer (eEOC). However, there is limited evidence regarding whether survival outcomes of laparoscopy are equivalent to those of laparotomy among patients with eEOC. The result of survival outcomes of laparoscopy is still controversial. The aim of this meta-analysis is to analyze the survival outcomes of laparoscopy versus laparotomy in the treatment of eEOC.

**Methods:**

According to the keywords, Pubmed, Embase, Cochrane Library and Clinicaltrials.gov were searched for studies from January 1994 to January 2021. Studies comparing the efficacy and safety of laparoscopy versus laparotomy for patients with eEOC were assessed for eligibility. Only studies including outcomes of overall survival (OS) were enrolled. The meta-analysis was performed using Stata software (Version 12.0) and Review Manager (Version 5.2).

**Results:**

A total of 6 retrospective non-random studies were included in this meta-analysis. The pooled results indicated that there was no difference between two approaches for patients with eEOC in OS (HR = 0.6, *P* = 0.446), progression-free survival (PFS) (HR = 0.6, *P* = 0.137) and upstaging rate (OR = 1.18, *P* = 0.54). But the recurrence rate of laparoscopic surgery was lower than that of laparotomic surgery (OR = 0.48, *P* = 0.008).

**Conclusions:**

Laparoscopy and laparotomy appear to provide comparable overall survival and progression-free survival outcomes for patients with eEOC. Further high-quality studies are needed to enhance this statement.

## Background

Ovarian cancer is one of the most common malignant tumors in gynecology, and its five-year survival rate is only about 46% after diagnosis, which has the highest death rate in gynecologic tumors [1, 2]. More than 95% of ovarian cancers are composed of epithelial ovarian cancer (EOC) with high morbidity and mortality compared with other rare pathological types of ovarian cancers. Despite the dismal prognosis with advanced-stage EOC, eEOCs tend to have an optimistic outcome with approximately 90% five-year survival rate if proper treatments are given [1, 3]. Early stage ovarian cancer is defined as an ovarian tumor limited to one or both ovaries with no evidence of local or distant spread at preoperative evaluation [4]. In view of the superior prognosis of eEOC, early diagnosis and precise treatment of EOC would be of great clinical significance for prognosis. Standard treatment for eEOC is comprehensive staging surgery and individualized postoperative chemotherapy. Laparotomy is generally recommended effective as a traditional approach for the management of EOC. In addition, after the first laparoscopic staging operation on ovarian malignancies was reported, laparoscopic staging procedure had been considered as an efficient and safe approach to assess and treat eEOC resembling that of laparotomy [5]. However, its application still remains controversial considering the unexpected tumor rapture or spillage, instrumental thermal injury, difficulty in tumor extraction and port-site metastasis etc. [6]. Some meta-analysis reported that laparoscopic surgery was associated with lower rates of complications, shorter postoperative hospital stays and similar in recurrence rate comparing to laparotomy [7, 8]. However, the sequential Cochrane Systematic Reviews revealed that there was no sufficient evidence to identify whether minimally invasive surgery can be a major clinical practice for early-stage eEOC [9]. Majority of the mentioned systematic reviews did not involve the overall survival evaluation. Therefore, this meta-analysis was designed to analyze the prognosis of eEOC in laparoscopy versus laparotomy especially in the respect of overall survival rate.

## Methods

### Search strategy

The study was conducted according to the meta-analysis of observational studies in epidemiology (MOOSE) guidelines. We performed a literature search using the keyword “laparoscopy”, “laparoscopic”, “robotic”, “minimally invasive surgery”, “early-stage ovarian cancer”, “early-stage adnexal cancer”, “stage I ovarian cancer”, “stage II ovarian cancer” in various combination from January 1994 to January 2021 in PubMed, Embase, Cochrane Library and Clinicaltrials.gov. Additionally, we also searched relevant references for articles. All studies were assessed by two investigators independently and any difference was settled by discussion. Studies in all languages were included.

### Study selection criteria and exclusion criteria

Articles comparing the survival outcomes of laparoscopy and laparotomy staging surgery with eEOC were considered appropriate for analysis. Studies were excluded via the following criteria: duplicate publications, abstracts, letters, case reports, reviews, single-arm studies and conference reports. Studies which did not focus on early-stage ovarian cancer were excluded. Studies involving other ovarian cancer types beyond EOC (e.g. germ cell tumors, sex cord stromal tumor, borderline tumors, etc.) were excluded. Studies involving laparoscopy surgery only for exploration not for therapeutic staging operation were excluded. Studies with insufficient data for estimating the HR and 95% CI were also excluded.

### Outcome measures

#### Primary outcomes

- Overall survival (4-5 years) (survival until death from any cause) (follow-up was decided by the initial studies)
- Progression-free survival (PFS) or disease-free survival (DFS) or recurrence-free survival (RFS) (for the purpose of this review, we have considered PFS, DFS and RFS to be the same endpoint)

#### Secondary outcomes

- Recurrence rate (4-5 years) (rate of recurrence)
- Upstaging rate (rate of postoperative upstaging)

### Data abstraction and quality assessment

The following data were extracted: baseline characteristics including age, BMI, stage of disease, how patients were found, reason for exclusion of participants, previous therapy and surgery, pre-operative preparation, level of training of surgeons, postoperative chemotherapy, follow-up, OS, PFS, upstaging rate, tumor rapture or spillage, methods of tumor extraction and port-site metastasis. The risk of bias in individual studies was assessed by Egger’s publication bias plot. Oxford center for evidence based medicine checklist for prognostic studies were used to evaluate the methodological quality.

### Statistical analysis

This meta-analysis was performed using Stata software (Version 12.0) and Review Manager (Version 5.2). The HR of the publications were obtained with Engauge Digitizer (Version 10.8). We used the Cochran Q test to evaluate the heterogeneity. Heterogeneity was used to evaluate the percentage of the variation in all studies. Because of inevitable clinical and methodologic diversity, inconsistency (I^2^) was adopted to quantify the effect of statistical heterogeneity. A value of 0% indicated no observed heterogeneity and a value more than 50% was considered substantial heterogeneity. If I^2^ > 50%, the random-effects model was adopted, otherwise, the fixed-effects model was used to pool the results. Review Manager (Version 5.2) was used to do the risk of bias graph and the risk of summary. A two-sided *P* < 0.05 was considered statistically significant.

## Results

### Study characteristics

**Fig. 1:**
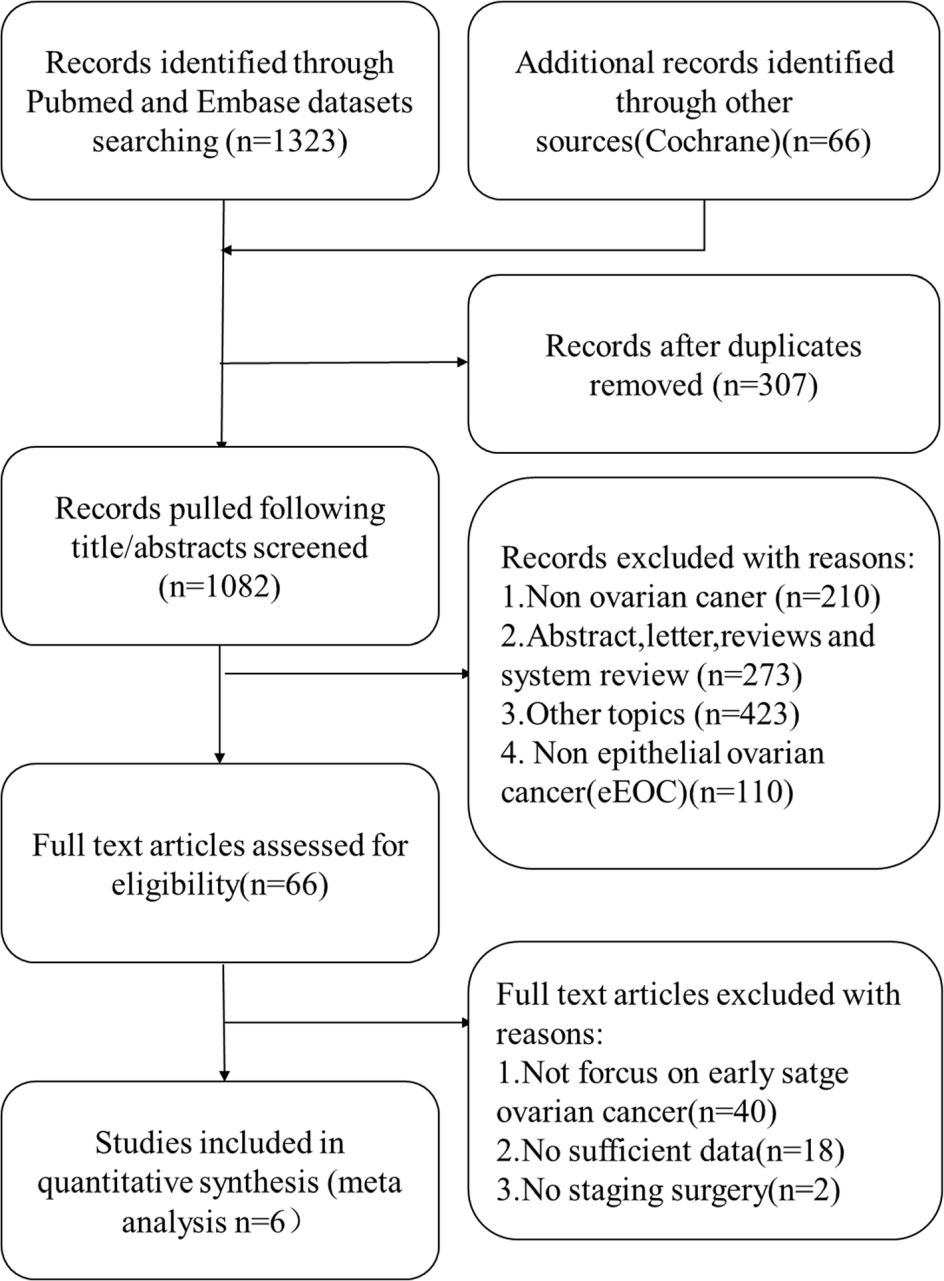
Flow diagram describing the research selection progress

The searching process was shown in Fig. 1. The number of records identified through PubMed, Embase and other sources was 1389. After screening titles and abstracts, 66 publications remained. In the 66 publications, 40 of them did not focus on early-stage ovarian cancer, 18 of them had insufficient data for estimating the HR and 95% CI, 2 of them involved laparoscopy surgery only for evaluation not for therapeutic staging operation purpose. Finally, 6 publications were selected for this meta-analysis incorporating data from 726 patients [10–15]. The selected studies were all retrospective cohort studies or case-control studies. Details of the 6 publications were shown in Table 1. All the included studies were published after 2014 from 4 countries, Italy for 2 studies, France for 1 study, Spain for 1 study, China for 2 studies. Mean duration of follow up ranged from 25.9–100 months, and the duration of the studies spanned over a decade (2002–2015). All patients involved in the articles were diagnosed with eEOC confirmed by histopathology intraoperative or postoperative and underwent a comprehensive staging operation. In Ditto et al.’s study, retroperitoneal staging was omitted in patients diagnosed with FIGO grade 1 mucinous tumor at the time of intraoperative frozen section [10]. Ditto et al., Xiong et al. and Merlier et al. included fertility-sparing procedures in the studies [10, 11, 15]. The median ages and BMI of patients ranged from 42 to 58 years old and 22 to 25.8 kg/m^2^ respectively. Three studies reported the method of samples extraction by using capsule bag. Only two studies reported ovarian tumor size, 6.75-8 cm in the laparoscopy group versus 10–14 cm in the laparotomy group respectively, which was of a statistically significant difference, and number of stage Ic1 (iatrogenic tumor rupture) ranged from 4.0% (2/50) to 15.4% (11/71) versus 6.8% (4/58) to 12.9% (4/31). However the whole tumor rupture or spillage rate was not mentioned in these studies. Number of pelvic-aortic lymph nodes dissected was 15–31 in the laparoscopy group and 13.5–61 in the laparotomy group respectively [10–15].

**Table 1:** Main characteristics of the selected researches

Study cohort	Study design	Year (study duration)	Study region	No. (LPS/LPT)	Age (years)	Outcome	Follow up (months)	BMI (kg/m^2^)	Tumor size (cm)(LPS/LPT)	Method of tumor extraction	No. of tumor spillage (LPS/LPT)	No. of lymph nodes removed (LPS/LPT)	No. of upstaging (LPS/LPT)	No. of adjuvant therapy (LPS/LPT)	No. of recurrence (LPS/LPT)	No. of port-site metastasis (LPS/LPT)
Lu, Q.	retrospective study	2016	China	42/50	LPS:54 (14–69)	OS	82 (16–152)	LPS:23.5 ± 4.0	NR	capsule bag	NR	20/22	9/10	12/15	5/6	0
		(2002–2014)			LPT:58 (25–76)			LPT:24.8 ± 3.2								
Minig, L.	retrospective comparative observational study	2016	Spain	50/58	LPS:52 (44.8–62.3)	OS/PFS	LPS:25.9 (11.2–38.5)	LPS:23.7 (21.8–27.2)	6.75 / 10.0	endo-bag	NR	15/13.5	12/8	32/34	1/7	0
		(2006–2014)			LPT:52 (44.8–65)		LPT:34.3 (28.4–47.8)	LPT:24.6 (21.8–28.5)								
Gallotta, V.	retrospective case-control study	2016	Italy	60/120	LPS:48 (24–73)	OS/PFS	38 (24–48)	NR	NR	NR	NR	16/ 18	NR	70%, similar between LPS/LPT	5/16	0
		(LPS:2007–2013;LPT:2000–2011)			LPT:55 (22–81)											
Ditto, A.	retrospective study	2017	Italy	50/50	LPS:46.0 ± 13.4	OS/PFS	LPS:49.5 ± 64	LPS:25.1 ± 3.4	NR	NR	NR	31/36	10/13	31/28	NR	0
		(2005–2015)			LPT:42.8 ± 11.2		LPT:52.6 ± 31.7	LPT:25.8 ± 5.1								
Xiong, W.	retrospective study	2017	China	71/31	LPS:47 ± 10	OS	50.5	LPS:23 ± 4	8/14	capsule bag	NR	18.1/15.5	7/2	67/59	11/4	0
		(2007–2014)			LPT:49 ± 10			LPT:22 ± 3								
Merlier, M.	retrospective study	2000–2018	France	37/107	LPS:56.3 ± 16.8	OS/PFS	LPS:24 (11–50)	LPS:23.8 ± 5.0	NR	NR	NR	28/67	NR	34/96	2/31	0
					LPT:56.2 ± 14.7		LPT:42 (22–66)	LPT: 25.4 ± 5.0								

*LPT* Laparotomy, *LPS* Laparoscopy, *BMI* Body Mass Index, *OS* Overall Survival, *PFS* Progression Free Survival, *NR* Not Report

### Risk of bias in included studies

**Fig. 2:**
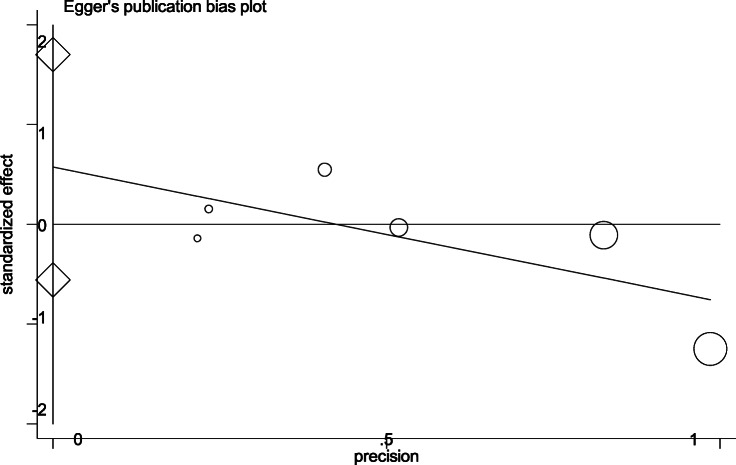
Egger’s publication bias plot of the studies included in this meta-analysis

**Fig. 3:**
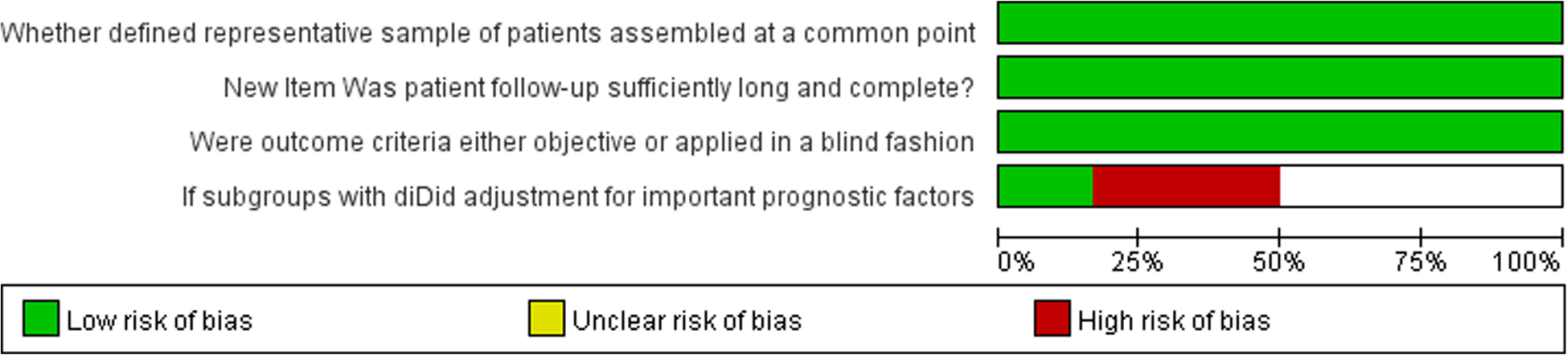
Risk of bias graph presenting authors’ judgements according to Oxford center for evidence based medicine checklist for prognostic studies about each risk of bias item as percentages across all included studies

**Fig. 4:**
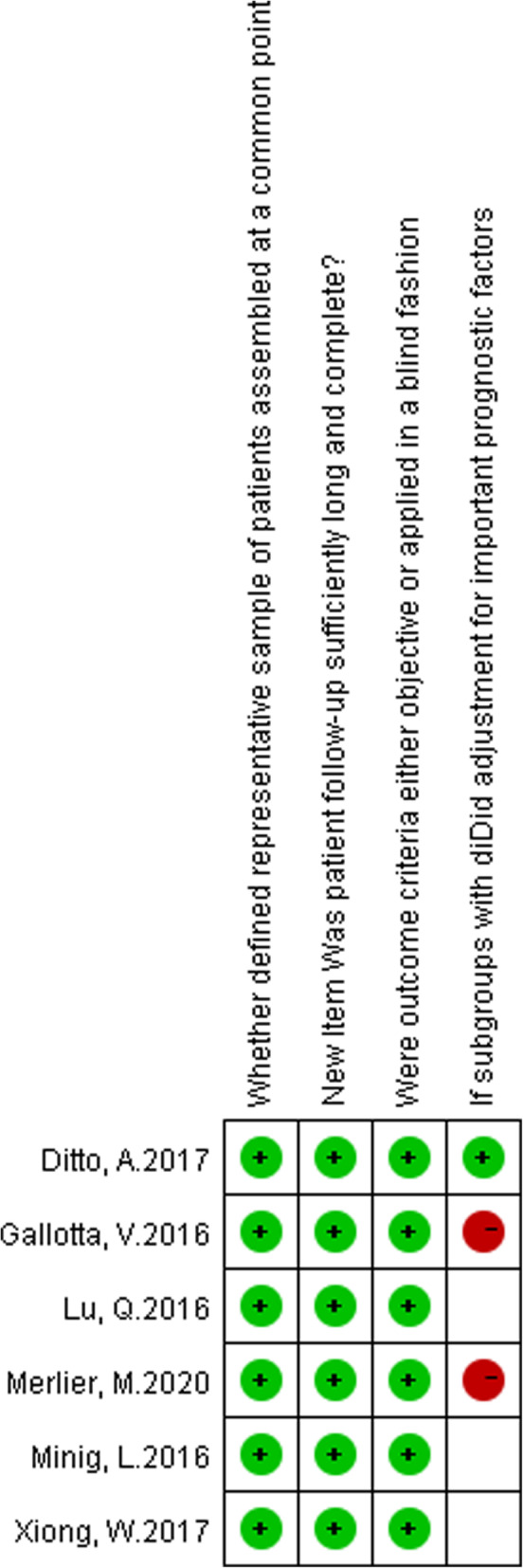
Risk of bias summary presenting authors’ judgements according to oxford center for evidence based medicine checklist for prognostic studies about each risk of bias item as percentages across all included studies

Egger’s publication bias plot of the studies included in this meta-analysis was notably symmetrical indicating that there was no obvious bias in publication shown in Fig. 2. According to Oxford center for evidence based medicine checklist for prognostic studies, the risk of bias graph was shown in Fig. 3, the risk of bias summary was shown in Fig. 4. A sensitivity analysis was not performed because of consistency in the risk of bias about the selected studies.

### Primary outcome

#### Overall survival

**Fig. 5:**
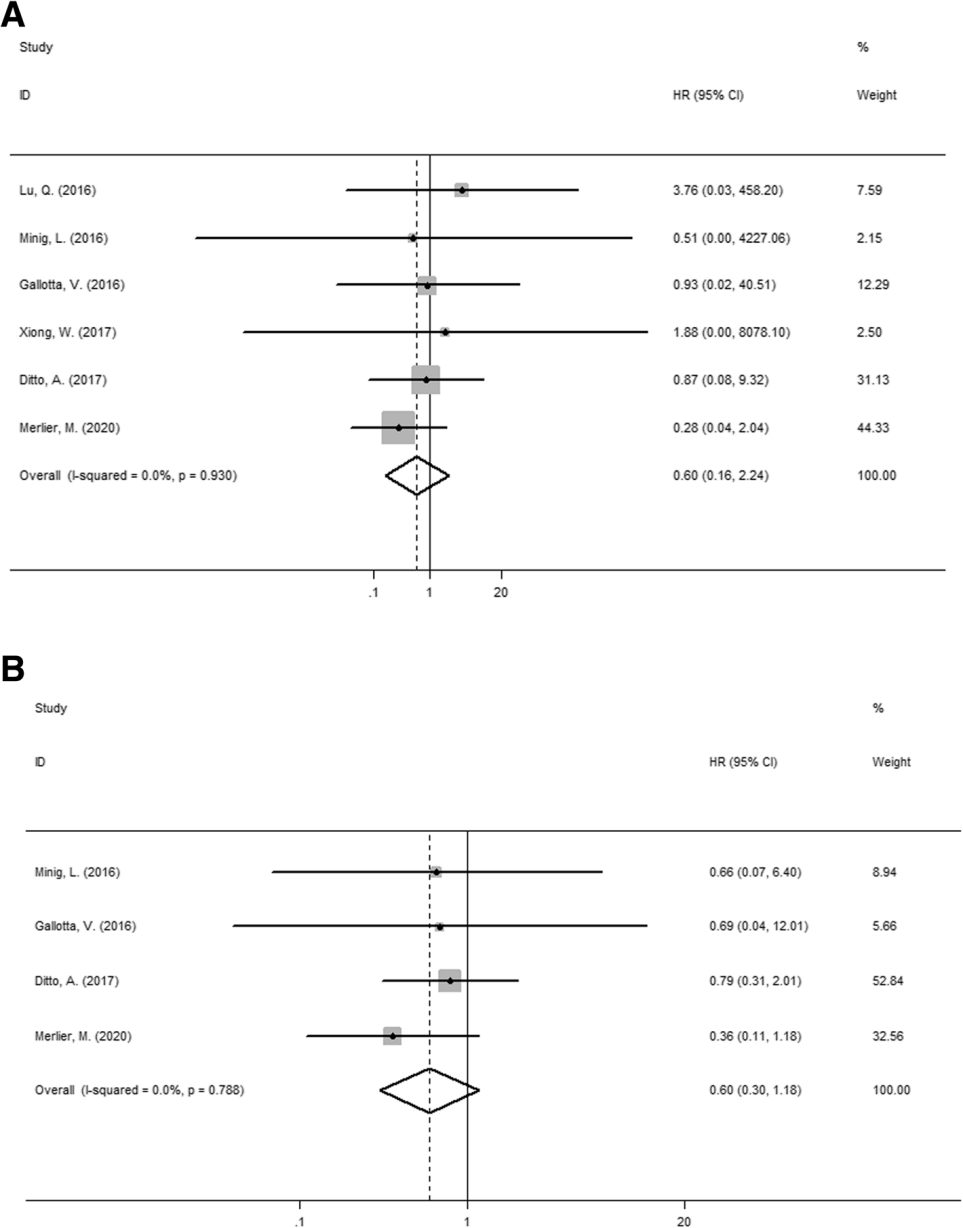
**a** Forest plot for the overall survival of laparoscopy versus laparotomy for patients with eEOC **b** Forest plot for the progression free survival of laparoscopy versus laparotomy for patients with eEOC

We analyzed the overall survival (4-5 years) of laparoscopy versus laparotomy for patients with eEOC in the 6 publications. The pooled results indicated that there was no difference in OS between two approaches (HR = 0.6, 95% CI: 0.16–2.24, I^2^ = 0.0%, *P* = 0.93 for heterogeneity, *P* = 0.446 shown in Fig. 5). In Lu et al.’s study, 5-year survival rates were 91.3% versus 88.4% in the laparoscopic group and laparotomic group respectively (*P* > 0.05). While, according to Xiong et al.’s study, 5-year overall survival rates were 86.7% versus 86.8% in laparoscopic groups and laparotomic groups, respectively (*P* = 0.874). Gallotta et al.’s study reported that 4-year OS rates were 92% in the laparoscopic group, 91% in the laparotomic group (*P* = 0.719). Merlier et al.’s study reported 5-year OS rates were 97.3% in the laparoscopic group, 79.8% in the laparotomic group. The other two studies did not mention accurate OS rate. However, Ditto et al.’s study reported after a mean (standard deviation) follow-up of 49.5 (64) versus 52.6 (81.8) months after laparoscopic and laparotomic surgery, there was no difference in survival outcomes. The similar results were indicated in Minig et al.’s study (the mean OS of 85.4 months versus 67 months in LPS and LPT (*p* > 0.05)).

#### Progression free survival

Data from 4 publications were extracted to analyze the progression-free survival (two studies did not provide PFS data). The pooled results indicated that there was no difference in PFS between two groups (HR = 0.6, 95% CI: 0.3–1.18, I^2^ = 0.0%, *P* = 0.788 for heterogeneity, *P* = 0.137, shown in Fig. 5). Gallotta et al.’s study reported that 4-year OS rates were 89% in the laparoscopic group, 81% in the laparotomic group (*P* = 0.651). The accurate PFS rate of patients were not mention in the other two studies. Ditto et al.’s study reported that after a mean (standard deviation) follow-up of 49.5 (64) versus 52.6 (81.8) months after laparoscopic and laparotomic surgery, there was no differences in survival outcomes. Minig et al.’s study reported the similar results (the mean PFS of 73.6 months versus 64.8 months in LPS and LPT (*P* > 0.05)).

### Secondary outcome

#### Recurrence rate

**Fig. 6:**
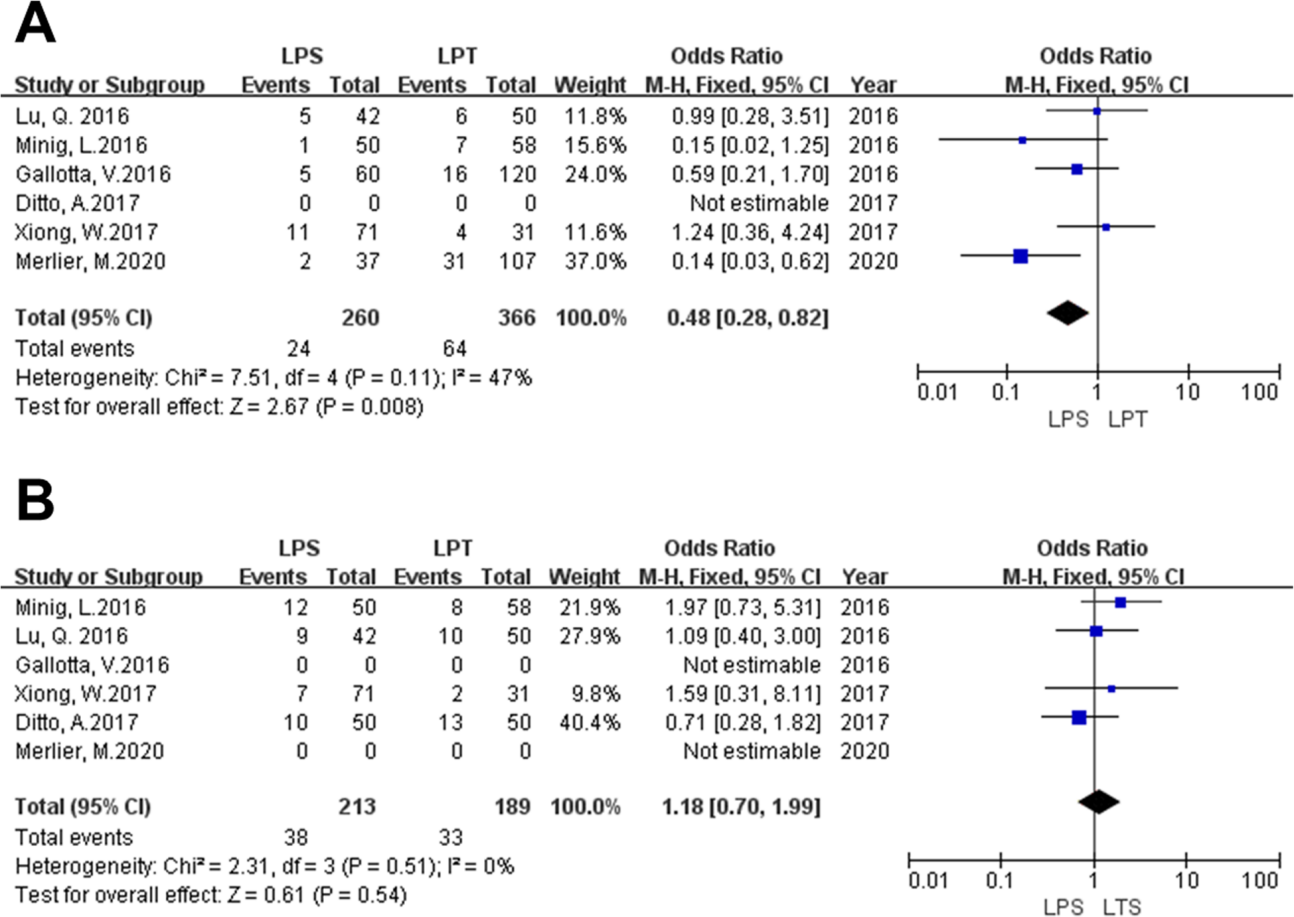
**a** Forest plot for the recurrence rate of laparoscopy versus laparotomy for patients with eEOC **b** Forest plot for the upstaging rate of laparoscopy versus laparotomy for patients with eEOC

Data from 5 publications were extracted to analyze the recurrence rate (one study did not provide recurrence rate data). The pooled results indicated that recurrence rate in laparoscopic surgery was lower than that in laparotomic surgery (OR = 0.48, 95% CI = 0.28–0.82, I^2^ = 47.0%, *P* = 0.11 for heterogeneity, *P* = 0.008, shown in Fig. 6). Gallotta et al.’s study reported that there were 8 peritoneum recurrence in laparotomic group verse 4 in laparoscopic group, 6 parenchymal recurrence in laparotomic group verse 1 in laparoscopic group, 2 lymph nodes recurrence in laparotomic group verse 0 in laparoscopic group. Minig et al.’s study reported there were 6 peritoneum recurrence in laparotomic group verse 5 in laparoscopic group, 1 lymph nodes recurrence in laparotomic group verse 1 in laparoscopic group. Merlier et al.’s study reported there were 16 peritoneum recurrence in laparotomic group verse 1 in laparoscopic group, 4 lymph nodes recurrence in laparotomic group verse 0 in laparoscopic group, 9 distant recurrence in laparotomic group verse 1 in laparoscopic group. The other 3 studies did not report the detail of recurrence.

#### Upstaging rate

Date of the upstaging rate were extracted from 4 publications (two studies did not provide upstaging rate data). The pooled results indicated that there was no difference in upstaging rate between two groups (OR = 1.18, 95% CI = 0.70–1.99, I^2^ = 0.0%, *P* = 0.51 for heterogeneity, *P* = 0.54, shown in Fig. 6). A higher upstaging rate in Lu et al.’s study (LPS versus LPT was 21.4% versus 20%) than that in Xiong et al.’s study (LPS versus LPT was 10% versus 6%).

## Discussion

This meta-analysis was focusing on the prognosis of laparoscopic surgery versus laparotomic surgery for patients with eEOC. After researching 1389 articles, no RCTs had been published focusing on the prognosis of laparoscopic surgery versus laparotomic surgery for patients with eEOC up to now. Due to the difficulty in recruiting sufficient patients and ethical issues, prospective studies had not been published either, only 6 existing retrospective studies met our criteria. The Cochrane review about this subject focusing on RCTs and prospective studies, did not find studies meet the criteria either [16]. The quality of retrospective studies was much worse than that of prospective studies. Based on the symptom of early-stage ovarian cancer was insidious and most patients were found in advanced stages, retrospective study was the only evidence available at present with no prospective studies, which may bring thinking to clinical research and treatment. Although retrospective studies had more risk of publication bias, quality assessment might down the risk. The quality assessment in the article showed a little risk of publication bias in the retrospective studies included. Besides, the inconsistency of retrospective studies could be solved by the strict definition, such as the standard of FIGO. In conclusion, this meta-analysis based on retrospective studies still provided some useful clinical information for the treatment of early stage ovarian cancer.

In 1994 Querleu and Leblanc created a precedent of surgical staging procedure for patients with ovarian or fallopian tube malignancies by using laparoscopic technique [5]. In the area of gynecologic oncology, laparoscopic surgery had been widely accepted by more and more surgeons and patients due to its advantage of minimal trauma and rapid recovery [17, 18]. However, its application still remained controversial because of the unpleasant complications such as the unexpected tumor rapture or spillage, instrumental thermal injury, difficulty in tumor extraction and port-site metastasis etc., which were of enormous importance to patients’ prognosis.

One of the most serious complications of laparoscopic surgery was port site metastasis which might be a crucial factor impacting the survival outcomes. Zivanovic et al. reported a port site metastases rate of 1.96% in 767 laparoscopic procedures among patients with ovarian cancer and all of the patients with port site metastases were in advanced stage [19]. In the study of Rutten et al., the port site metastases rate was 3%, and all of these patients were with advanced ovarian cancer [20]. The Cochrane database review indicated that port site metastases mostly occurred in the setting of advanced disease [9]. A recent report by Carboni et al. indicated that port site metastases rate of patients with ovarian cancer was only 0.3% [21]. All studies included in this meta-analysis did not have port site metastases, mostly because a significant percentage of patients remained in early stage and surgeons had a rising awareness about port site metastases. There are several hypotheses for the cause of port-site metastasis, including direct transmission of the device and the “chimney stack effect” in which the tumor cells spread along trocar [22]. CO_2_ pneumoperitoneum might be an influencing factor of the hypothesis of “chimney stack effect” in laparoscopic surgery. CO_2_ pneumoperitoneum during laparoscopic surgery might promote inflammation and tumor progression. However, the study by Abu-Rustum et al. indicated that comparing between the second-look laparotomy versus laparoscopy treating 289 patients with ovarian cancer or primary peritoneal cancer, CO_2_ pneumoperitoneum did not appear to reduce the overall survival [23]. Prophylaxis of port site metastases can have various causes such as using wound protectors, minimizing unnecessary manipulation, using an endo-bag to remove the large specimen and closure of the incision at the end of the procedure. Effective prevention can reduce the incidence of port site metastasis.

Upstaging rate might be another factor which affected the outcome of OS and PFS. Firstly, intraoperative tumor rupture and spillage might be a major reason which affected the upstaging rate. The review by Tantitamit et al. concluded that the rate of intraperitoneal spillage among patients with ovarian cancer treating with laparoscopy was 1.3 to 37.5%. The main factor influenced intraperitoneal spillage might be the size of tumor. Larger tumors might be more likely to rupture [24]. The studies included in this study did not reported the information about intraperitoneal spillage. The risk of tumor spillage was not only limited to laparoscopic surgery, one study reported tumor rupture in laparoscopy which was similar to that in laparotomy for patients with early-stage ovarian cancer (10.5% versus 12.1%, respectively; *P* = 1.000) [25]. The study of Dembo et al. did not find a significant relationship between intraoperative rupture and prognosis for patients with eEOC [26]. However, in the study of Bakkum-Gamez et al. indicated that in stage I ovarian cancer intraoperative capsule rupture might lead a higher risk of recurrence and death [27]. The clinical significance of tumor rupture during surgery is still unclear. Xiong et al. reported the 5-year overall survival rate in stage I c1 (iatrogenic rupture) eEOC patients with laparoscopy and laparotomy were 78.8 and 75.0% respectively, which is of no significant difference [11]. The prognostic value of intraoperative tumor rupture needs more evaluation based on large-scale RCTs in patients with eEOC.

Secondly, more lesions can be found by adequate exploration and surgical excision during the operation which might affect the upstaging rate. The critical factor for management of ovarian cancer is primary surgery with complete excision of macroscopic and palpable lesions in the pelvis and abdomen. It has been thought that completion staging was technically difficult to be attainable via laparoscopy, nevertheless, with the superiority in optical magnification and refinement of laparoscopic techniques, laparoscopic surgery performed by expert surgeons can achieve a complete assessment and treatment equivalent to laparotomy [25]. However, lack of tactile assessment might lead missing of invisible disease concealed in patients’ abdomen or pelvis during laparoscopic procedures. Lymph nodes status might be a crucial predictor of survival outcome. The number of lymph nodes removed can be considered as a sign of adequate surgery. A lot of studies have proved that lymph node dissection of laparoscopy for patients with eEOC is already adequate and safe [28, 29]. All studies included in this meta-analysis have compared lymph node yields between two approaches for patients with eEOC. The results indicated that the number of total lymph node retrievals was comparable between two groups. It might be an important that the staging quality between two groups was not inferior. The present meta-analysis indicated that there was no difference in upstaging rate between two groups. Additionally, extensive preoperative evaluation such as pelvic and abdominal CTs, PET-CTs, can be used to detect early metastases so that they can be resected precisely and in a timely manner.

The results indicated that there was no difference in 4-5 years OS, PFS and postoperative upstaging rate respectively between two surgical approaches for eEOC. However, recurrence rate in laparoscopic surgery was lower than that in laparotomic surgery. Sensitivity analysis of recurrence rate showed the study of Merlier et al. was the reason of heterogeneity [15]. Excluded this study, there was no difference in recurrence rate between two surgical approaches with eEOC. The major reason of this might be the difference of operative procedure. The study of Merlier et al. was analyzed in various factors, the major difference about high recurrence rate might be related to it included fertility-sparing procedures. Some studies introduced that fertility-sparing procedure was a viable surgery for women who had fertility requirements with early-stage ovarian cancer [30, 31]. However, some studies against this proposition for the recurrence rate of early-stage ovarian cancer with fertility-sparing procedure was higher than that with traditional comprehensive surgery. The study of Bentivegna et al. concluded that the recurrence rate of grade 3 or stage IC3 or clear cell tumor treating with fertility-sparing procedure had higher recurrence rate. The prognosis of fertility-sparing procedure for grade 3 or stage IC3 or clear cell tumor was still unclear [32]. The study of Merlier et al. (included in this meta-analysis) did not report the number of fertility-sparing procedure in two surgical approaches, laparotomic group had more samples, which might have more fertility-sparing procedure. To sum up, fertility-sparing procedure for women with early-stage ovarian cancer might be the reason for the lower recurrence rate in laparoscopic surgery in this study. And the safety and efficiency of fertility-sparing procedure in eEOC still need more studies. In addition, the size of tumor might be another reason for the higher recurrence rate in laparotomic surgery. There were two studies included in this meta-analysis reported the size of tumor. In the laparotomy group, the tumor size were much lager than that in the laparoscopic group. As we all known, the larger tumor, the higher rupture of tumor. Unfortunately, there was no information about the rupture in included studies. More studies were needed for the point.

This meta-analysis reviewed clinical researches comparing the survival outcome of laparotomy and laparoscopy for patients with eEOC through January 2021. Our results are valuable for surgeons, given the comparable survival outcomes between two surgical approaches. The main limitation of our study is that the number of included studies is small relatively and all of the inclusive publications are retrospective in design. The reason of this is that there has been no prospective studies or RCTs published up to now about the prognosis in laparotomic surgery versus laparoscopic surgery for patients with eEOC. An ongoing RCT (NCT02686463) seeks to provide definitive comparison of outcome data between laparoscopic and laparotomic surgeries in eEOC patients. The result of this RCT might provide more convincing data. Additionally, eEOC tends to have a favorable prognosis with the 5-year survival rate after comprehensive surgical treatment reaching 90% [1]. In many studies, there was no death case during the follow-up time, so further longer follow-up researches are needed.

## Conclusions

In conclusion, this meta-analysis indicated that laparoscopy and laparotomy appear to provide comparable survival outcomes, and laparoscopy might be an efficient and safe procedure for patients with eEOC. Further high-quality studies are needed, especially, randomized controlled trials (RCTs).

## Data Availability

All data is available in this paper.

## Abbreviations

EOC: Epithelial ovarian cancer
eEOC: Early-stage epithelial ovarian cancer
OS: Overall survival
PFS: Progression-free survival
NOS: Newcastle-Ottawa Scale
HR: Hazard ratio
CI: Confidence interval
LPT: Laparotomy
LPS: Laparoscopy
